# Bridge Nucleic Acid/DNA Gapmers as Potential Inhibitors of Bacterial Gene Expression by Multiple Antisense Mechanisms: An In Vitro Study

**DOI:** 10.3390/molecules30244721

**Published:** 2025-12-10

**Authors:** Angel J. Magaña, Kimberly Phan, Jesse A. Lopez, Maria S. Ramirez, Marcelo E. Tolmasky

**Affiliations:** Center for Applied Biotechnology Studies, Department of Biological Science, California State University Fullerton, Fullerton, CA 92821, USA; angel.magana@csu.fullerton.edu (A.J.M.); kkphan@csu.fullerton.edu (K.P.); lopezj146@csu.fullerton.edu (J.A.L.); msramirez@fullerton.edu (M.S.R.)

**Keywords:** antisense, bridge nucleic acid, RNase P, RNase H, aminoglycoside, antibiotic resistance

## Abstract

Antisense inhibition of gene expression is usually achieved using nuclease-resistant oligonucleotide analogs that promote mRNA degradation through RNase H or RNase P, or by steric hindrance of translation. Bridge nucleic acids (BNAs) are nucleotide analogs available in a few chemical variants. We evaluated gapmers composed of an oligodeoxynucleotide flanked by BNA residues in a BNA_5_-DNA_8_-BNA_4_ configuration, using the available variants: the original locked nucleic acid (LNA; 2′-O,4′-methylene bridge), cET (2′-O,4′-ethyl bridge), cMOE (2′-O,4′-methoxyethyl bridge), and BNANC (2′-O,4′-aminomethylene bridge). These gapmers were tested in vitro for their ability to induce cleavage of the model *aac(6′)-Ib* mRNA. All gapmers complementary to a previously identified region suitable for interaction with antisense oligomers induced RNase H-mediated degradation. Instead, only the LNA-containing gapmer also elicited RNase P-dependent cleavage, demonstrating dual RNA- and DNA-mimicking capability. In vitro coupled transcription–translation assays using cell lysates or reconstituted systems confirmed inhibition of expression and ruled out steric hindrance as the mechanism. In contrast, gapmers targeting the ribosome-binding site strongly inhibited expression by steric hindrance. These findings demonstrate that LNA-containing gapmers can exert their effects through multiple mechanisms, depending on the targeted mRNA region, thereby supporting their potential for synergistic inhibition of gene expression.

## 1. Introduction

Antisense technologies are a group of approaches designed to interfere with harmful biological processes by reducing the expression of target genes using complementary oligonucleotides or oligonucleotide analogs [1,2,3,4,5,6,7]. In the case of eukaryotic systems, these technologies can also be utilized to modify splicing of pre-mRNA and modify expression or correct defects [1,4,8,9]. Although these technologies face numerous challenges, such as stability, toxicity, delivery, and cellular penetration, many antisense drugs have been introduced to the market, and many others are in development [2,3,7,10]. However, most drugs approved or in advanced clinical trials do not target bacterial pathogens. Despite considerable past and ongoing efforts [11,12,13,14,15], developing effective antisense pharmacological tools against bacterial infections remains an unmet challenge [3]. Interference with gene expression mediated by antisense compounds can mostly occur by steric hindrance, where the antisense impedes the binding or progression of the ribosome along the mRNA or through eliciting cleavage of the target mRNA [3,5,6]. Various strategies to induce steric hindrance or mRNA degradation have been explored. Two particularly intensive research areas focus on turning off prokaryotic genes by promoting the degradation of the target mRNA through recruiting endogenous RNases like RNase H or RNase P [2,3,10]. The former alternative takes advantage of the property of the endonuclease RNase H, an enzyme characterized by its ability to cleave RNA when it is in a duplex with DNA in a non-sequence-specific manner [16]. The latter utilizes the ribozyme RNase P, which, in conjunction with a protein cofactor, cleaves duplex RNA:RNA in a structure-specific but sequence-nonspecific manner [17]. In this case, the antisense molecule is known as “external guide sequence” (EGS), and the general approach is known as EGS technology [17,18,19].

Viable antisense drugs must resist nucleases that could degrade them before reaching their target. This property can be achieved by constructing oligomers totally or partially composed of nucleotide analogs [2,20,21,22]. However, the oligomers must retain their ability to bind the complementary sequence, and the endogenous enzyme must recognize the duplex as substrate. If RNase H is the intended cleavage agent, the analog must resemble DNA when interacting with the target. On the other hand, if the desired compound should behave as an EGS, i.e., cleavage occurs via RNase P digestion, the antisense bound to the target must resemble a duplex RNA:RNA with the appropriate structure. A way to improve the efficiency of antisense-mediated gene silencing could be to recruit both endogenous RNases to degrade the target mRNA.

**Figure 1:**
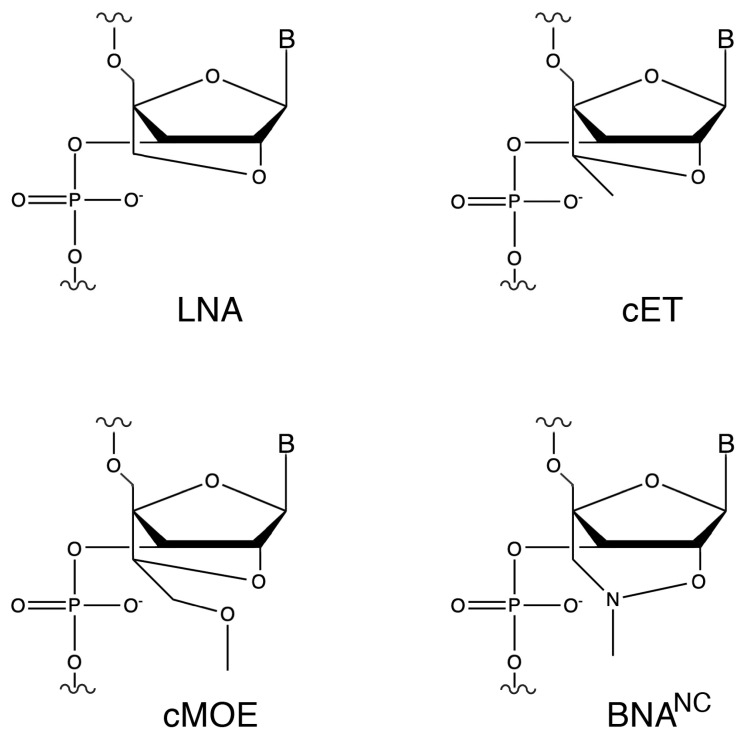
Chemical structures of ribonucleotide analogs. LNA, 2′-O-4′-methylene locked nucleic acid; cET, 2′-O,4′-ethyl bridge nucleic acid; cMOE, 2′-O,4′-methoxyethyl bridge nucleic acid, BNA^NC^, 2′-O,4′-aminomethylene bridge nucleic acid.

To test this possibility, we aimed to design oligonucleotide analogs capable of eliciting degradation of the target mRNA by both RNase H and RNase P. As a model, we used the *aac(6′)-Ib* gene, which encodes the aminoglycoside 6′-*N*-acetyltransferase type Ib [AAC(6′)-Ib]. This enzyme is responsible for the inactivation of amikacin in most amikacin-resistant Gram-negative bacteria [23,24]. Inhibition of expression of *aac(6′)-Ib* would enable the use of amikacin and other aminoglycosides to treat severe multidrug-resistant infections [25]. Our previous research showed that hybrid oligomers containing locked nucleic acid (LNA) and deoxyribonucleotide residues in a gapmer configuration were promising analogs for recruiting RNase P to cleave target mRNA molecules and turn off expression of bacterial genes [22,26,27]. Since the early development of LNAs, the first bridge nucleic acid (BNA) analogs, characterized by a methylene group linking the 2′-oxygen and the 4′-carbon of the ribose residue (2′-*O*,4′-methylene-β-d-ribofuranosyl nucleotides), other BNA derivatives have been introduced or are in development (Figure 1) [22,28].

In this work, we tested gapmers consisting of an oligodeoxynucleotide flanked by different BNA analogs to determine their ability to elicit RNase H- and RNase P-mediated mRNA cleavage. Our results showed that gapmers composed of a stretch of LNA residues flanking an oligodeoxynucleotide are most efficient in eliciting degradation by both RNases. Furthermore, we showed that they can also reduce gene expression by steric hindrance depending on the location of the complementary region in the target mRNA.

## 2. Results

Previous comprehensive studies aimed at identifying nuclease resistant analogs that function as efficient EGSs, i.e., they elicit RNase P cleavage of a target RNA molecule, showed that hybrid oligomers containing LNA and deoxyribonucleotide residues were the only active constructs among those tested. RNase P recruitment depends on formation of an appropriate pre-tRNA structure rather than binding strength alone [18]. Duplex stability is significantly affected by LNA, each LNA residue increases Tm by ~1 to 8 °C against DNA and ~2 to 10 °C against RNA [29]. Therefore, the number of LNA and deoxyribonucleotide residues in the EGS is crucial to determine the strength of interaction. However, for an LNA/deoxyribonucleic oligomer to be an efficient EGS, not only is the ratio of residues essential, but also the configuration. Our prior structure activity relationship studies using homogeneous or hybrid oligomers, including multiple nucleotide analog classes and hybrid configurations demonstrated that efficient RNase P recruitment and cleavage of the target RNA requires a gapmer with an optimal ratio and arrangement of analogs and deoxyribonucleotide residues, with the chemistry and configuration LNA_5_-DNA_8_-LNA_4_ producing the best results [26,27,30]. Building on that information, we designed a series of isosequential gapmers incorporating the currently available BNA analog variations: LNA, 2′-O-4′-methylene locked nucleic acid; cET, 2′-O,4′-ethyl bridge nucleic acid; cMOE, 2′-O,4′-methoxyethyl bridge nucleic acid, BNA^NC^, 2′-O,4′-aminomethylene bridge nucleic acid (Table 1 and Figure 1).

**Figure 2:**
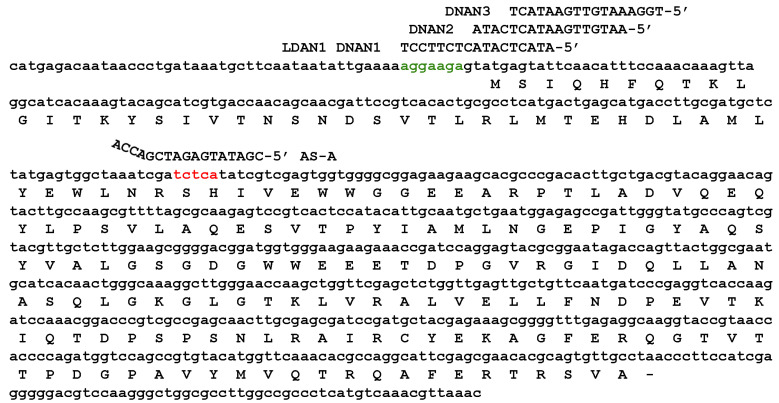
Nucleotide sequences of the *aac*(*6′*)*-Ib* mRNA and antisense oligomers. Complete sequence of the coding strand corresponding to the mRNA used in this work showing the AAC(6′)-Ib amino acid sequence, the Shine-Dalgarno (green), and the region A, accessible to interact with antisense oligomers (red) as described in Sarno et al. [32]. AS-A, general nucleotide sequence of oligomers used in this study.

The gapmers are complementary to a region of the *aac(6′)-Ib* gene that was first identified as available for pairing with antisense oligoribonucleotides and elicit RNase P activity (region A, identified in red in Figure 2) [31,32]. The gapmers used had the BNA_5_-DNA_8_-BNA_4_ configuration, which was previously shown to be the most effective in eliciting mRNA cleavage by RNase P and inhibiting expression of *aac(6′)-Ib* in a recent study comparing multiple configurations [26]. All gapmers used in this study were designed to include the sequence ACCA at the 3ʹ-end to enhance substrate recognition by RNase P. The ACCA sequence corresponds to the conserved RCCA motif found at the 3ʹ-end of pre-tRNAs, the natural substrates of RNase P. This sequence pairs with the complementary UGG sequence located within the P15-loop of M1 RNA, the catalytic subunit of the RNase P ribozyme. Then, its presence helps the EGS-target RNA duplex resemble the pre-tRNA structure facilitating RNase P recruitment and cleavage [33].

**Figure 3:**
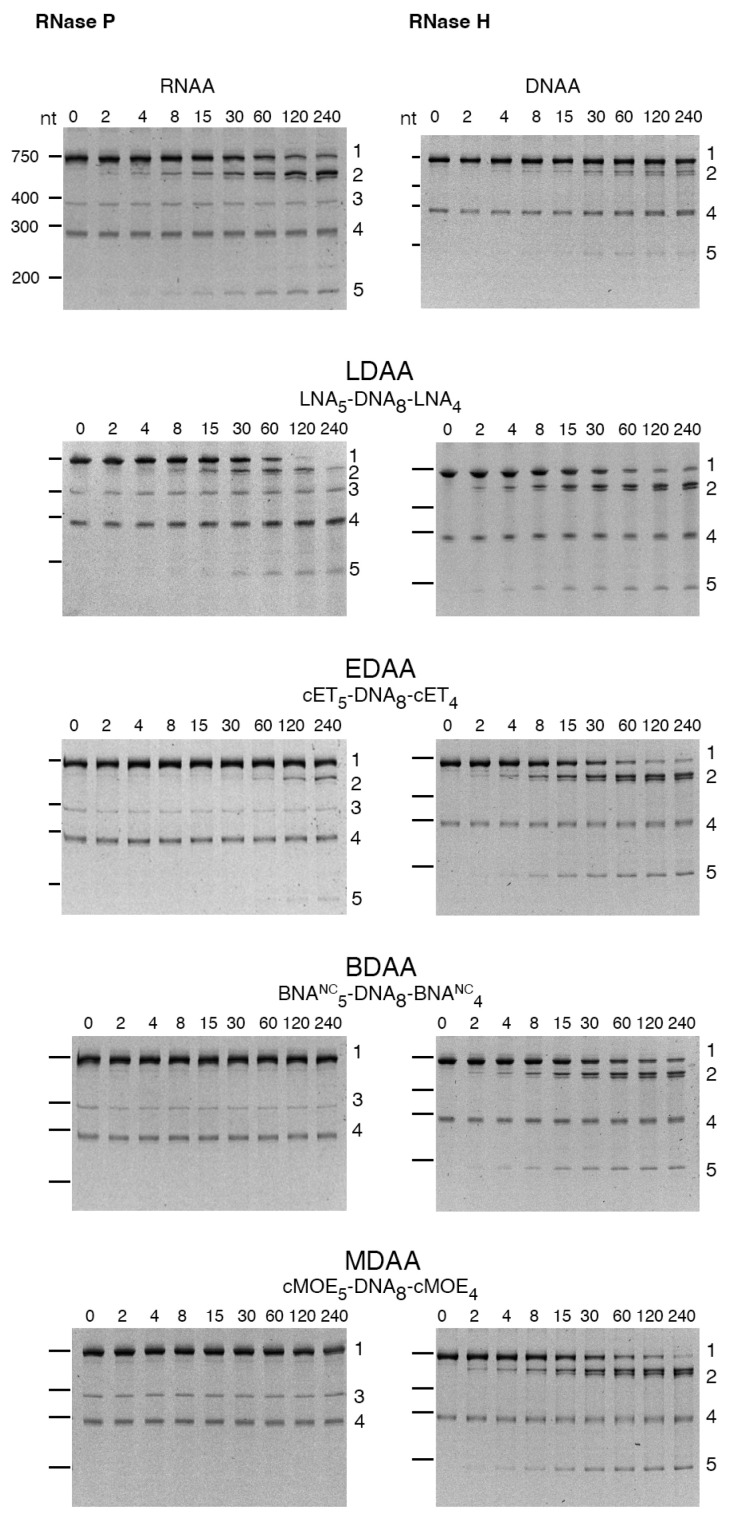
RNase H or RNase P cleavage of *aac(6′)-Ib* mRNA-oligonucleotide analog duplex. The cleavage reactions were carried out as described in Section 4. Reactions were stopped by adding 2 volumes of loading buffer (15 mM Tris, 0.75 mM EDTA, 6 M urea). The products were analyzed using 6% denaturing Tris-borate EDTA-polyacrylamide gel electrophoresis. The gels were stained at room temperature with shaking for 30 min in a 1× Biotium PAGE GelRed solution and visualized on an ultraviolet transilluminator. Incubation times were 0, 2, 4, 8, 15, 30, 60, 120, and 240 min (left to right lanes after the molecular size standards, loaded on the leftmost well). The numbers to the right identify the nature of the compounds: 1, uncleaved mRNA; 2, 3′-end cleavage product; 3, M1, RNA component of RNase P; 4, unidentified; 5, 5′-end cleavage products. The numbers to the left indicate the nucleotide lengths (nt) of the molecular size standards.

The cleavage of the *aac(6′)-Ib* mRNA was assessed in the presence of RNase P or RNase H using the gapmers constructed with different BNAs (Table 1). Figure 3 shows that RNase H exhibited activity with all gapmers tested. Similar levels of cleavage were observed when using LNA-, cMOE-, and cET-containing gapmers (LDAA, MDAA, and EDAA). A lower activity was found in the case of BDAA, the BNA^NC^-containing gapmer (Figure 3). Conversely, significant cleavage by RNase P was observed only with the LDAA gapmer, which elicited complete mRNA degradation during the incubation period (Figure 3). In all cases, including those lacking RNase P, we observed an approximate 290-nucleotide band of unknown origin present in the original sample. This band has been observed previously and is most likely a secondary transcription product generated during the synthesis of the *aac(6′)-Ib* mRNA.

The results of these assays demonstrate that hybrid antisense analogs with an appropriate chemical structure can act as DNA or RNA analogs, effectively generating appropriate substrates for RNase H and RNase P, respectively. The BNA-DNA-BNA gapmers are hybrid molecules that simultaneously present RNA-like and DNA-like conformational features. The central deoxyribonucleotide fragment provides a DNA-like segment within the RNA/gapmer duplex, a requirement for RNase H activity. On the other hand, the BNA flanking regions have an RNA-like structure due to the 2′-O,4′-C bridge [34]. Thus, the gapmers tested in this study can be viewed as a DNA-like central portion flanked by RNA-like segments.

RNase H cleaves the phosphodiester bonds of RNA in DNA/RNA duplexes with little or no dependency on their nucleotide sequence [35], while RNase P requires a pre-tRNA structure for recognition and cleavage. Therefore, a chimeric duplex containing both DNA-like and RNA-like features could, in principle, support recruitment of either enzyme. However, in our experiments, among the BNA variants tested, only LNA-containing gapmers supported RNase P cleavage suggesting that LNA provides the structural features most compatible with formation of an RNase P substrate. This dual activity could enhance antisense efficacy by designing molecules that recruit both RNase H and RNase P to degrade the target mRNA.

**Figure 4:**
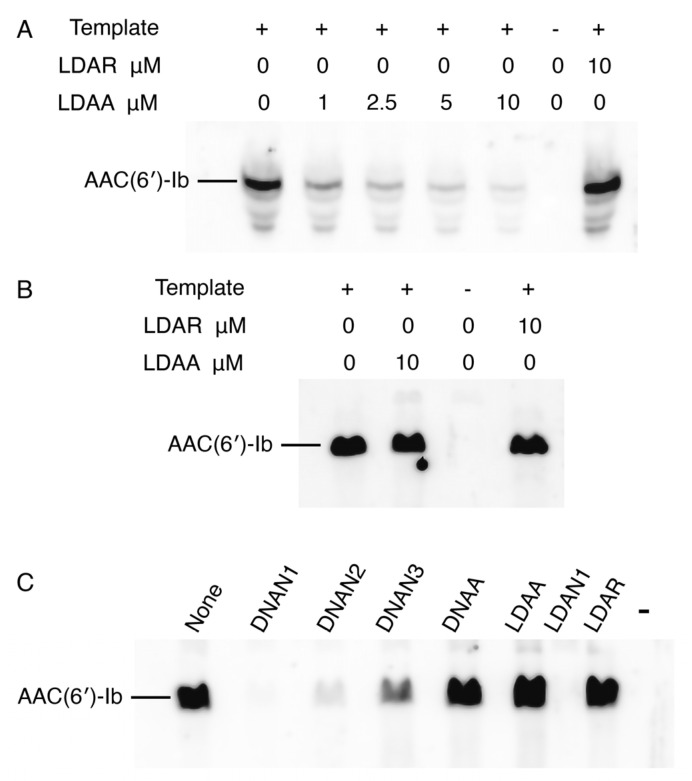
Inhibition of *aac(6′)-Ib* expression. In all cases, the template was plasmid pAMND201 DNA, which carries the *aac(6′)-Ib* gene. Reactions were carried out as described in Section 4 and the protein was detected by western immunoblot. (**A**) Coupled transcription–translation reactions using cell lysates in the presence of increasing concentrations of LDAA. Control reactions were carried out with no antisense (leftmost lane), with addition of LDAR, a gapmer with a random sequence (rightmost lane), or without template and antisense oligomers (next to the rightmost lane). (**B**,**C**) Coupled transcription–translation reactions using a reconstituted system lacking RNase H and RNase P. (**B**) the experimental reaction is shown in second from left lane, control lanes are reactions lacking antisense (leftmost lane), template and antisense second lane from the rightmost), or with the addition of LDAR (rightmost lane). (**C**) reactions performed in the presence of various antisense molecules (10 μM), as indicated above each lane. The rightmost lane corresponds to a reaction where addition of template and antisense were omitted.

To assess the correlation between target mRNA degradation in enzymatic assays and inhibition of AAC(6′)-Ib protein synthesis, we performed experiments using coupled transcription–translation reactions in a cell lysate or a reconstituted system. This approach permits sidestepping the limitations of cell penetration by the antisense compounds. The cell lysates contain endogenous RNase H and RNase P, whereas the reconstituted extract lacks both enzymes. Reactions conducted in a cell lysate supplemented with the recombinant plasmid pAMND201, which carries *aac(6′)-Ib*, resulted in robust expression of the AAC(6′)-Ib protein (Figure 4A). The addition of the LDAA oligomer inhibited expression in a dose-dependent manner. In contrast, a control gapmer, LDAR, with the same chemical configuration but a random nucleotide sequence, had no effect (Figure 4A). In a second assay, coupled transcription–translation was performed using the reconstituted system. As shown in Figure 4B, the addition of LDAA did not interfere with AAC(6′)-Ib expression. Expression levels were comparable to those observed in reactions without antisense added or with LDAR, indicating that in the absence of RNase H and RNase P, LDAA has no inhibitory effect. This result ruled out steric hindrance, suggesting that mRNA cleavage is the only LDAA mechanism of inhibition of *aac(6′)-Ib* expression when the antisense molecule targets region A.

The absence of a steric hindrance effect when targeting region A prompted us to evaluate the impact of antisense molecules directed at regions encompassing or adjacent to the ribosomal binding site (RBS), highlighted in green in Figure 2. Previous studies have shown that antisense molecules of a different chemical nature than those tested here, complementary to sequences within or near the RBS or translation start site, act by preventing binding and assembly of the ribosome [3,36]. Coupled transcription–translation reactions using the reconstituted system showed that an oligodeoxynucleotide complementary to a region including the RBS (DNAN1, Figure 2) inhibited *aac(6′)-Ib* expression (Figure 4C). The inhibitory effect decreased as the sequence targeted by the antisense molecule was shifted farther from the RBS (DNAN2 and DNAN3; Figure 2 and Figure 4C), indicating that the target location on the mRNA is critical for determining both the mechanism and the extent of inhibition. A gapmer isosequential to DNAN1, LDAN1, was tested in the reconstituted system, and, unlike those targeting region A, there was strong inhibition by steric hindrance (Figure 4C). As expected, a gapmer with a random nucleotide sequence had no effect on translation (Figure 4C). These results demonstrate that gapmers composed of LNA residues flanking an oligodeoxyribonucleotide, in the configuration tested here, can interfere with gene expression through any of the three mechanisms examined, depending on the location of the complementary region within the target mRNA.

## 3. Discussion

Antisense inhibition of gene expression has been investigated as the basis of a therapeutic strategy for several decades with limited success. Aside from fomivirsen, an antiviral antisense agent that inhibits replication of human cytomegalovirus, approved for clinical use in 1998 but later discontinued for commercial reasons [37,38], antisense medicines did not reach the market until the second decade of the twenty-first century. Moreover, those approved or currently in the pipeline are intended primarily for diseases other than bacterial infections [1,3]. Ongoing efforts to develop antisense drugs for bacterial pathogens include strategies aimed at either essential genes, functioning as actual antibiotics, or resistance genes, serving as adjuvants to existing antibiotics [3]. The main mechanisms of antisense-mediated inhibition explored to date involve either steric hindrance or recruitment of endogenous ribonucleases such as RNase H or RNase P. When inhibition is achieved via RNase P-mediated cleavage of the target mRNA, the approach is referred to as EGS technology. Because resistance to nucleases is a requirement for viable antisense drugs, several groups have studied the action of oligonucleotide analogs. Peptide nucleic acids and phosphorodiamidate morpholino oligomers have been used to inhibit bacterial growth, most probably by steric hindrance [39,40,41]. Attempts to design EGSs composed partially or entirely of analogs have generally been unsuccessful, except for gapmers consisting of an oligodeoxynucleotide flanked by LNA residues [30]. In particular, gapmers with LNA_5_-DNA_8_-LNA_4_ configuration demonstrated the highest activity in the limited tests performed to date [26,27]. LNAs were the first of the BNA family of analogs, characterized by a methylene bridge between the 2′-oxygen and the 4′-carbon of the ribose ring. The development of additional BNA variants permitted us to build on this finding, assessing the activity of other gapmers with identical configuration. The goal of the study was to identify gapmers capable of eliciting target mRNA degradation through the recruitment of both RNase H and RNase P. While all the gapmers tested promoted RNase H-mediated cleavage, only the LNA-containing gapmer induced substantial RNase P-mediated degradation of the *aac(6′)-Ib* mRNA. This dual activity of LDAA, eliciting both RNase H and RNase P cleavage to act on the target transcript, was a novel finding, underscoring its potential as a potent antisense inhibitor of gene expression. Given that the recruitment of endogenous RNase P by EGS molecules is the basis of EGS technology, our findings with the LNA_5_-DNA_8_-LNA_4_ gapmer suggest a promising extension of this approach. We propose the extended methodology, named EGS-PH technology, that combines the actions of RNase P and RNase H to enhance mRNA degradation and gene silencing efficiency. The results also showed that small changes in the chemical structure of the BNA analog can result in substantial changes in activity.

To circumvent the challenge of cellular uptake, we evaluated the activity of the LNA gapmer using a lysate-based cell-free coupled transcription–translation system. LDAA inhibited *aac(6′)-Ib* expression in a dose-dependent manner. In contrast, a reconstituted in vitro system lacking RNase H and RNase P showed no inhibition, indicating that LDAA does not interfere with ribosome progression. On the other hand, a second LNA-containing gapmer targeting the RBS strongly inhibited expression via steric hindrance. These findings indicate that the gapmers studied here can act through different mechanisms depending on the region to which they are complementary. They also suggest that combining gapmers with distinct modes of action may lead to synergistic antisense effects. Although these assays are still in early development and require validation with additional targets, we are optimistic that strategies integrating EGS-PH technology with gapmers that act via steric hindrance hold significant promise for achieving robust and specific inhibition of undesirable gene expression, potentially opening new avenues for antisense-based therapeutic strategies.

## 4. Materials and Methods

### 4.1. Bacterial Strain, Plasmids, and Oligonucleotides

*Escherichia coli* TOP10 F^−^ *mcrA* Δ(*mrr-hsd*RMS-*mcr*BC) Φ80*lac*ZΔM15 Δ*lac*X74 *rec*A1 *ara*D139 Δ(*ara-leu*)7697 *gal*U *gal*K *rps*L(Str^R^) *end*A1 *nup*G was used for recombinant cloning and plasmid isolation. Plasmid pJA’, which includes the *Escherichia coli rnpB* gene downstream of the T7 promoter [42], was used as template to generate the amplicon utilized to synthesize the M1 RNA component of RNase P. The primer sequences to generate the amplicon are shown in Table 1. Plasmid pRHC5 was generated by inserting a DNA fragment containing the *rnpA E. coli* gene downstream of the T7 promoter in pT7-5 [31]. This plasmid was used to express and purify the protein C5 component of RNase P. Plasmid DNA isolation was carried out using the QIAspin miniprep or midi kit (QIAGEN, Germantown, MD, USA). The plasmid pAMND201, obtained by cloning a pJHCMW1 fragment containing the *aac(6′)-Ib* gene (nucleotides 7111 to 7971, accession number AF479774) into the pCR2.1 vector, was used to synthesize the *aac(6′)-Ib* mRNA in vitro (MEGAscript T7, Thermo Fisher Scientific, Waltham, MA, USA) and for coupled in vitro transcription-translation. The nucleotide sequence of the primers to generate the amplicon to be inserted into the pCR2.1 clong vector are shown in Table 1. The sequence and configurations of the oligomers tested as antisense are shown in Table 1. The analogs used were LNA, 2′-O-4′-methylene locked nucleic acid; cET, 2′-O,4′-ethyl bridge nucleic acid; cMOE, 2′-O,4′-methoxyethyl bridge nucleic acid; and BNA^NC^, 2′-O,4′-aminomethylene bridge nucleic acid (Figure 1). Gapmers and oligoribonucleotides were purchased from BioSynthesis Inc., Lewisville, TX, USA. Oligodeoxynucleotides were purchased from IDT.

### 4.2. RNase P Preparation

M1 RNA was synthesized in vitro using a commercial kit (MEGAshortscript T7, Thermo Fisher Scientific, Waltham, MA, USA) following the recommendations of the supplier using an amplicon containing the *rnpB* gene under the control of a T7 promoter as a template [31]. The amplicons were purified by running on 1% agarose gel electrophoresis and eluted from the gel using the Zymoclean Gel DNA Recovery kit (Zymo Research, Irvine, CA, USA). The cofactor C5 protein was purified after expressing it from an *E. coli* strain that harbors a plasmid containing *rnpA* under the control of the T7 promoter. Both components were combined to generate the RNase P holoenzyme. RNase H was commercially available (NEBiolabs, Ipswich, MA, USA).

### 4.3. In Vitro RNase P and RNase H Assays

RNase H and RNase P-mediated cleavage of *aac(6′)-Ib* mRNA was carried out by preincubating 2 pmol *aac(6′)-Ib* mRNA and 10 pmol of the antisense at 37 °C for 30 min in a volume of 3 μL. Then, all 3 μL were mixed with a solution containing enzyme (0.25 pmol of M1 RNA, 70 pmol of C5 protein for RNase P, 20 mM HEPES-KOH pH 8, 400 mM ammonium acetate, and 10 mM magnesium acetate, or 1 unit RNase H in 1.4× RNase H buffer) that had been preincubated at 37 °C for 15 min in a final volume of 7 μL. The reactions were incubated at 37 °C for the times indicated. Reactions were stopped by adding 2 volumes of loading buffer (15 mM Tris, 0.75 mM EDTA, 6 M urea). The products were analyzed using 6% denaturing Tris-borate EDTA-polyacrylamide gel electrophoresis. The gels were stained for 30 min in a 1× Biotium PAGE GelRed solution for 30 min with shaking at room temperature and visualized on an ultraviolet transilluminator.

### 4.4. Coupled In Vitro Transcription-Translation

Coupled transcription-translation reactions were carried out with the S30 T7 High-Yield Protein Expression System (Promega, Madison, WI, USA) and the PUREfrex 2.0 (GeneFrontier Corporation, Kashiwa, Chiba, Japan) as recommended by the supplier. The former system uses cell lysates, while the latter is a reconstituted cell-free protein synthesis system composed of amino acids, NTPs, tRNAs, RNA polymerase, translation factors, purified ribosomes, and other small-molecule compounds and proteins but lacking RNase H and RNase P [43]. All reactions were performed in the presence of 500 ng plasmid pAMND201 DNA template with the additions indicated in each case. The final volumes of each reaction were 25 and 20 μL in the case of assays done with cell lysates and with the reconstituted extracts, respectively. The reactions were incubated at 37 °C for 1 and 2 h, respectively, with shaking (200 rpm). Then, SDS gel-loading solution was added, and the products were separated by sodium dodecyl sulfate-polyacrylamide gel electrophoresis (SDS-PAGE), and the synthesized AAC(6′)-Ib was subsequently detected by immunoblotting. After SDS-PAGE, the proteins were electrophoretically transferred to nitrocellulose paper using the Mini Trans-Blot cell (BioRad, Hercules, CA, USA). The blots were rinsed in phosphate-buffered saline with Tween detergent (PBST) and blocked overnight at 4 °C in PVDF Blocking Reagent (Toyobo, Osaka, Japan). The membrane was then rinsed at room temperature in PBST for 15 min with shaking, rinsed twice in PBST for 5 min with shaking, and incubated for 1 h at room temperature with anti-AAC(6′)-Ib serum (10 μL of the adsorbed antiserum mixed with 10 ml Can Get Signal solution 1 (Toyobo)). After removal of the antiserum solution, the blots were washed three times with PBST for 5 min at room temperature with shaking. The blots were then incubated for 1 h at room temperature with a solution containing horseradish peroxidase-labeled protein A (Sigma, St. Louis, MO, USA) in Can Get Signal solution 2 (Toyobo, Osaka, Japan). After removal from this solution, the blots were rinsed three times in PBST for 5 min at room temperature with shaking, followed by incubation in a solution containing SuperSignal™ West Pico PLUS Chemiluminescent Substrate (Thermo Fisher Scientific, Waltham, MA, USA) for 10 min with shaking. Chemiluminescence was detected on the Omega Lum™ C Imaging System 81-12110-00 (Gel Company, San Francisco, CA, USA).

## Figures and Tables

**Table 1:** Oligonucleotides used in this work.

Name	Sequence (5′-3′)	Chemistry
M1 amplicon F	TTGTAATACGACTCACTATAGGGGAAG	DNA
M1 amplicon R	AGGTGAAACTGACCGATAAG	DNA
*aac(6′)-Ib* mRNA F	GCAAGCTTTAATACGACTCACTATAGCATGAGACAATAACCCTGATAAATGCTTC	DNA
*aac(6′)-Ib* mRNA R	GTTTAACGTTTGACATGAGGGC	DNA
RNAA	rCrGrArTrArTrGrArGrArTrCrGrArCrCrA	RNA
DNAA	CGATATGAGATCGACCA	DNA
LDAA	[^L^C][^L^G][^L^A][^L^T][^L^A]TGAGATCG[^L^A][^L^C][^L^C][^L^A]	LNA_5_-DNA_8_-LNA_4_
BDAA	[^B^C][^B^G][^B^A][^B^T][^B^A]TGAGATCG[^B^A][^B^C][^B^C][^B^A]	BNA^NC^_5_-DNA_8_-BNA^NC^_4_
EDAA	[^E^C][^E^G][^E^A][^E^T][^E^A]TGAGATCG[^E^A][^E^C][^E^C][^E^A]	cET_5_-DNA_8_-cET_4_
MDAA	[^M^C][^M^G][^M^A][^M^T][^M^A]TGAGATCG[^M^A][^M^C][^M^C][^M^A]	cMOE_5_-DNA_8_-cMOE_4_
LDAR	[^L^G][^L^C][^L^T][^L^G][^L^A]CTGAAATG[^L^A][^L^C][^L^C][^L^A]	LNA_5_-DNA_8_-LNA_4_
DNAN1	ATACTCATACTCTTCCT	DNA
DNAN2	AATGTTGAATACTCATA	DNA
DNAN3	TGGAAATGTTGAATACT	DNA
LDAN1	[^L^A] [^L^T] [^L^A] [^L^C] [^L^T]CATACTCT[^L^T] [^L^C] [^L^C] [^L^T]	LNA_5_-DNA_8_-LNA_4_

^L^N, LNA; ^B^N, BNA^NC^; ^E^N, cET; ^M^N, cMOE. The T7 promoter nucleotide sequence is underlined.

## Data Availability

Data is contained within the article, further inquiries can be directed to the corresponding author.
